# P-2166. Fusariosis in Hematologic Malignancies in the Era of Antifungal Prophylaxis

**DOI:** 10.1093/ofid/ofaf695.2329

**Published:** 2026-01-11

**Authors:** Paulina Vega, Pooja Bhattacharyya, Thomas M Kuczmarski, Regina Lengermann, Lisa So, Leah H Yoke, Hayden Z Smith, Joshua Lieberman, David Fredricks, Steven A Pergam

**Affiliations:** University of Washington/ Fred Hutch Cancer Center, Seattle, WA; Fred Hutch/University of Washington, Seattle, Washington; University of Washington, Seattle, Washington; FHCC, Seattle, Washington; Fred Hutch/University of Washington, Seattle, Washington; University of Washington; Fred Hutch Cancer Research Center, Seattle, WA; University of Washington, Seattle, Washington; University of Washington, Seattle, Washington; Fred Hutchinson Cancer Research Center; University of Washington, Seattle, WA; Fred Hutchinson Cancer Center; University of Washington, Seattle, WA

## Abstract

**Background:**

Patients receiving myelosuppressive chemotherapy or undergoing hematopoietic cell transplant (HCT) for hematologic malignancies (HM), are disproportionately affected by invasive fusariosis (IF). We performed a 10-year retrospective review to characterize the epidemiology of IF in the era of mold-active triazole (MAT) prophylaxis.

**Figure:**
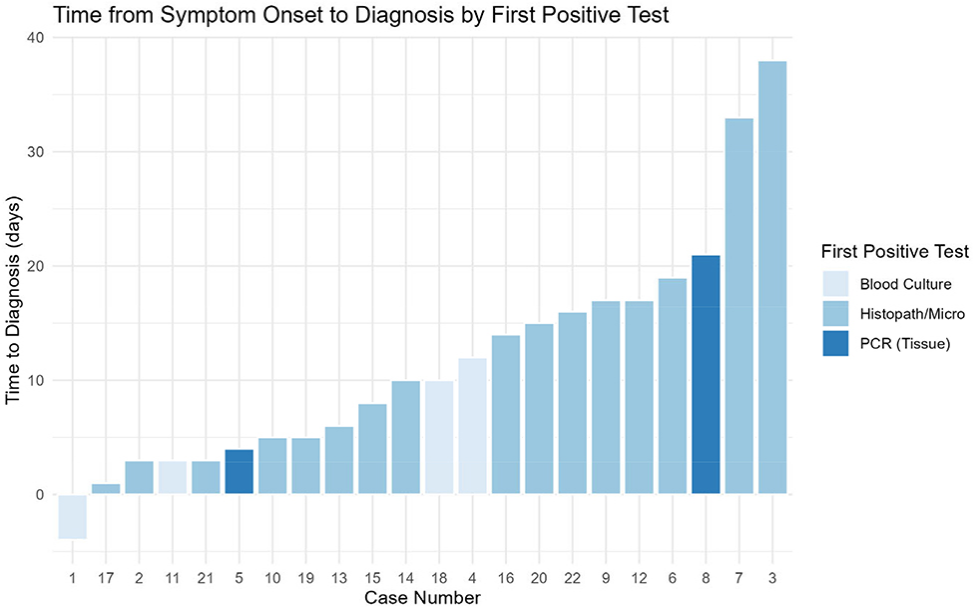

**Figure:**
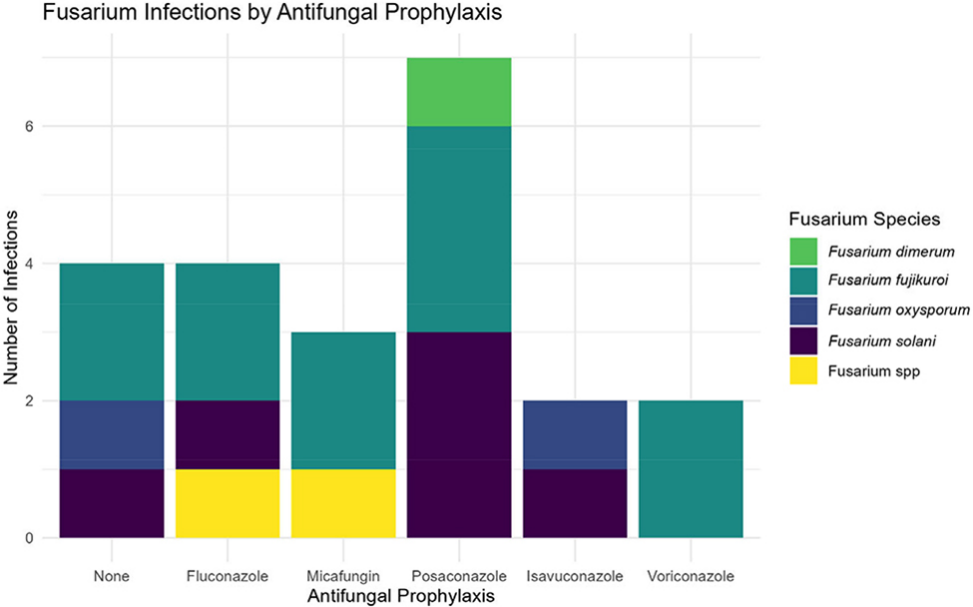

**Methods:**

To be included, patients had to be ≥18 years of age and diagnosed with IF at our tertiary cancer center between July 2015 and June 2024. All cases met international definitions for proven/probable fusarium infections including positive microbiologic or molecular testing. Demographics, presenting symptoms, diagnosis, treatments and outcomes were evaluated by 2 independent reviewers.

**Figure:**
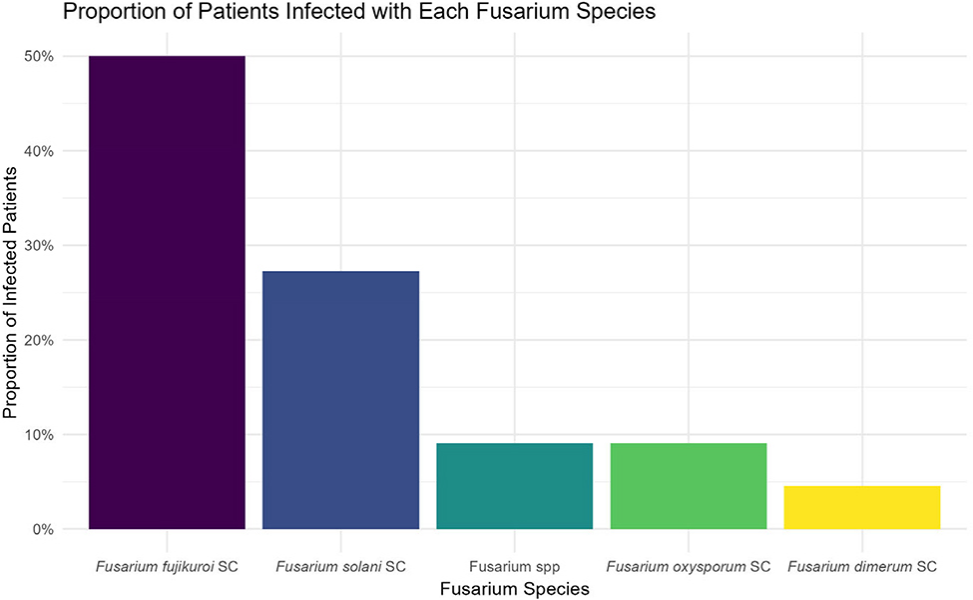

**Figure:**
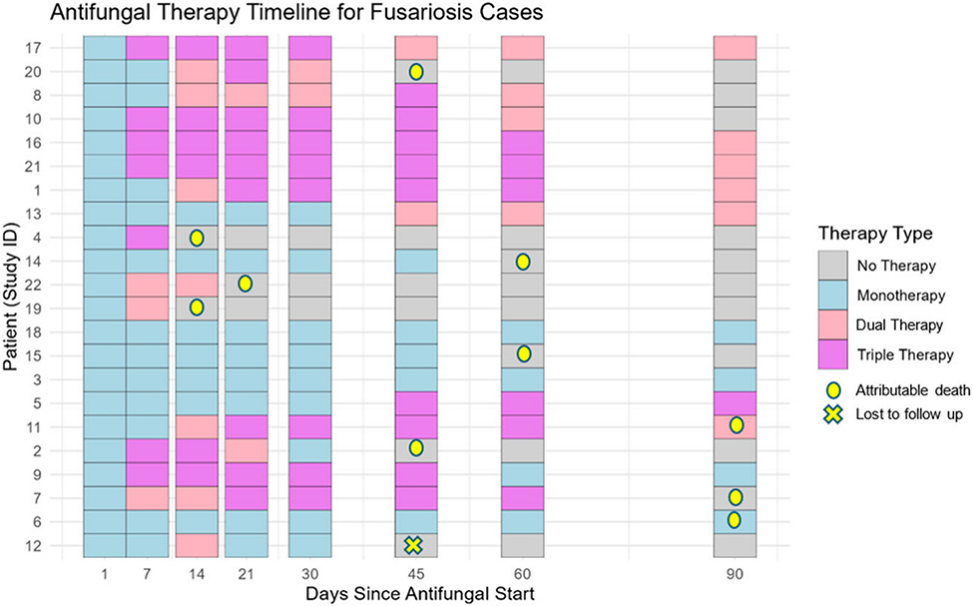
This graph depicts antifungal therapy type (monotherapy, dual, triple) at day 1, 7, 14, 21, 30, 45 , 60 and 90. Yellow circles show cases in which death was attributable to invasive fusariosis. Yellow cross shows a case who survived but was lost to follow up.

**Results:**

Twenty-two cases of IF were identified. A total of 16 (73%) had AML; 15 (68%) were undergoing re-induction chemotherapy and 9 (41%) had a prior HCT. Most were neutropenic at diagnosis 21 (96%), with a median duration of 48 days (range 9-93). Fever was the most common symptom (77%), followed by skin nodules (68%). Eighteen patients (82%) had pulmonary nodules on chest CT, despite only 4 (14%) reporting respiratory symptoms. Fungemia was detected in 7 cases (31%). Initial diagnosis was made via skin biopsy (12), sinus sampling (6), and blood cultures (4); all were negative for serum galactomannan (GM). Median time to diagnosis from symptom onset was 10 days (range 1-38) (Fig.1). Of the 18 (82%) on antifungal prophylaxis most received MAT (11 [61%]) (Fig 2). *F. fujikuroi* species complex (SC) were the most common species identified (50%), followed by *F. solani* SC (27%) (Fig 3). Liposomal amphotericin B was the most frequent initial empiric therapy; most transitioned to dual or triple regimens within 7 days (Fig 4). Adjunctive therapies included G-CSF (11), granulocyte infusions (9), and surgery (9). Ten deaths (45%) were attributed to IF.

**Conclusion:**

Breakthrough IF remains a persistent concern for patients being treated for HMs in the era of MAT. Despite reports of potential utility of GM in IF, all cases in our cohort were negative. The observed diversity of antifungal treatment highlights the need for more data on optimal antifungal management. These data confirm that IF remains a life-threatening infection in HM patients with prolonged neutropenia.

**Disclosures:**

Joshua Lieberman, MD, PhD, Anavasi Diagnostics: in kind support for reagents and equipment|Qiagen: in kind support for reagents and equipment|Roche: in kind support for reagents and equipment|ThermoFisher: in kind support for reagents and equipment David Fredricks, MD, BD: Royalty|Seres Therapeutics: Advisor/Consultant Steven A. Pergam, MD, MPH, F2G: Site PI for clinical trial|Global Life Technologies, Inc.: Grant/Research Support|Mundipharma: Site PI for clinical trial|Symbio: Site PI for clinical trial

